# Association of Number of Indoor Tanning Salons With Neighborhoods With Higher Concentrations of Male-Male Partnered Households

**DOI:** 10.1001/jamanetworkopen.2019.12443

**Published:** 2019-10-04

**Authors:** Rebecca Chen, J. Aaron Hipp, Lily Morrison, Lisa Henriksen, Susan M. Swetter, Eleni Linos

**Affiliations:** 1 Weill Cornell Medical College, New York, New York; 2 Department of Dermatology, Stanford University Medical Center and Cancer Institute, Palo Alto, California; 3 Center for Geospatial Analytics, North Carolina State University, Raleigh; 4 Stanford Prevention Research Center, Stanford School of Medicine, Palo Alto, California; 5 Dermatology Service, Veterans Affairs Palo Alto Health Care System, Palo Alto, California; 6 Stanford Health Research and Policy, Stanford School of Medicine, Palo Alto, California

## Abstract

**Importance:**

Both indoor tanning and skin cancer are more common among sexual-minority men, defined as gay and bisexual men, than among heterosexual men. Convenient access to indoor tanning salons may influence use patterns.

**Objective:**

To investigate whether indoor tanning salons are disproportionately located in areas with higher concentrations of gay men.

**Design, Setting, and Participants:**

This cross-sectional study used geographic information systems to integrate census data and business location data obtained from ArcGIS and Google Maps for the 10 US cities with the largest lesbian, gay, bisexual, and transgender populations in 2010, ie, Los Angeles, California; Chicago, Illinois; San Francisco, California; Seattle, Washington; San Diego, California; Dallas, Texas; Phoenix, Arizona; Washington, DC; Portland, Oregon; and Denver, Colorado. The association of indoor tanning salon locations with proportions of gay men, using the concentration of male-male partnered households as a proxy measure for the latter, was examined. Data analysis was performed in October 2018.

**Exposures:**

Census tracts with at least 1%, 5%, or 10% male-male partnered households, adjusting for median household income, percentage young women, and percentage non-Hispanic white residents.

**Main Outcomes and Measures:**

Presence of 1 or more indoor tanning salons within census tracts.

**Results:**

Across the 10 cities and 4091 census tracts in this study, there were 482 823 unmarried partnered households, of which 35 164 (7.3%) were male-male. The median (interquartile range) percentage of male-male partnered households per census tract was 0% (0%-10.6%). Odds of indoor tanning salon presence in areas with at least 10% male-male households were more than twice those of areas with less than 10% male-male households (odds ratio, 2.17; 95% CI, 1.59-2.97). When sensitivity analyses using a 1-mile euclidian buffer around each tanning salon were conducted, this association remained significant (odds ratio, 2.48; 95% CI, 2.14-2.88). After adjusting for median household income, percentage young women, and percentage non-Hispanic white residents, the odds of an indoor tanning salon being within 1 mile of a census tract with at least 10% male-male households remained twice that of census tracts with less than 10% male-male households (odds ratio, 2.00; 95% CI, 1.71-2.35).

**Conclusions and Relevance:**

In this study, indoor tanning salons were more likely to be located near neighborhoods with higher concentrations of male-male partnered households, possibly contributing to the disproportionate use of indoor tanning by sexual-minority men.

## Key Points

**Question:**

Are indoor tanning salons more likely to be located in neighborhoods with higher proportions of gay men?

**Findings:**

In this cross-sectional study of 10 US cities with the largest proportion of lesbian, gay, bisexual, and transgender populations, the odds of indoor tanning salons being located in census tracts with higher concentrations of male-male partnered households were approximately 2-fold higher than in areas with lower concentrations of male-male partnered households. After sensitivity analyses, the association remained significant.

**Meaning:**

The results of this study suggest that tanning salons may be more common in areas with higher populations of male-male partnered households in US cities, possibly contributing to the disproportionate use of indoor tanning by sexual-minority men.

## Teaser

This cross-sectional study investigates whether indoor tanning salons are disproportionately located in areas with higher concentrations of sexual-minority men, defined as gay and bisexual men, using male-male partnered households as a proxy measure.

## Introduction

The burden of skin cancer in the United States continues to increase, with indoor tanning causing more than 450 000 basal and squamous cell cancers and more than 10 000 melanomas each year.^1^ Compared with heterosexual men, sexual-minority men (SMM), defined as gay and bisexual men, are estimated to have a 6-fold higher lifetime prevalence of tanning bed use and more than 2-fold higher lifetime prevalence of skin cancer.^2^ In addition, skin cancers are more common and more aggressive in persons living with HIV, which disproportionately affects SMM.^3^ Sexual-minority men report multiple reasons for seeking indoor tanning, including appearance, community, pleasure, and convenience.^4^ However, the association of the physical environment in which SMM live, including convenience, accessibility, and cost of indoor tanning, with the use of indoor tanning salons has not been addressed.

The built environment—that is, the humanmade surroundings that provide the space in which people live and work—influences health and contributes to health disparities.^5^ Neighborhoods influence health behaviors through socioecological qualities, including the availability of goods and services and community values.^6^ Based on an ecological model, persons living in neighborhoods with higher densities of indoor tanning salons may be more likely to engage in indoor tanning than persons living in neighborhoods with lower densities of indoor tanning salons because of the accessibility of and opportunities and reminders to use the salons.^7^ For example, indoor tanning salon locations are associated with areas that have more concentrated populations of groups more likely to participate in indoor tanning, including non-Hispanic white individuals and women aged 15 to 24 years.^8^ This cross-sectional study investigated whether indoor tanning salons are disproportionately located in neighborhoods with higher concentrations of gay men.

## Methods

### Data Collection

We conducted a cross-sectional study of the 10 cities in the United States with the largest lesbian, gay, bisexual, and transgender (LGBT) populations based on 2010 US Census data for concentration of same-sex couple households. The cities were Los Angeles, California; Chicago, Illinois; San Francisco, California; Seattle, Washington; San Diego, California; Dallas, Texas; Phoenix, Arizona; Washington, DC; Portland, Oregon; and Denver, Colorado.^9^ Stanford University determined this study to be exempt from institutional review board oversight and informed consent because it analyzes public, deidentified, secondary data. This report follows the Strengthening the Reporting of Observational Studies in Epidemiology (STROBE) reporting guideline for cross-sectional studies.

We identified indoor tanning salon locations in October 2018 by geographic coordinates using ArcGIS Business Analyst version 6.1 (Esri), which provides large and representative data sets, including business locations, and allows for the inclusion of additional data. To increase the chances of identifying all possible tanning salon locations, we cross-referenced indoor tanning salon locations identified using ArcGIS Business Analyst with search results on the Google Maps application programming interface platform version 3.34 (Google), which allows users to search business locations by keyword. Keywords searched on both databases included *tanning salon*, *indoor tanning*, and *tanning bed*. We excluded businesses that did not offer UV radiation–based indoor tanning by accessing business websites and confirming services offered.

There are multiple ways to calculate the concentration of same-sex couples, and they are highly correlated.^10^ The 2010 US Census did not ask about individual sexual identity, orientation, or same-sex married households. Therefore, to measure the concentration of SMM in a given area, we calculated the percentage of male-male partnered households as a fraction of the total number of unmarried partnered households in each census tract. This variable (collected by the US Census Bureau) refers specifically to partnered male-male households and does not include men living together as roommates. This method is similar to that used in other studies present in the literature.^10,11^ In a study by Walther and Poston,^10^ which developed 4 different indexes for determining the density of gay and lesbian people within a neighborhood, ratios using male-male households as the numerator were shown to be highly reliable in predicting the concentration of gay couples in a given area. To further assess our proxy variable for accuracy in measuring the concentration of SMM residents, we confirmed that the neighborhoods with the highest concentrations of male-male households were in 2 well-known LGBT neighborhoods (the Castro District of San Francisco and the West Hollywood area of Los Angeles). Additionally, we conducted a sensitivity analysis by calculating the percentage of male-male partnered households as a fraction of the total number of households (all married and unmarried partnered households) in each census tract to evaluate whether inherent differences in unmarried partnered households and married households (such as age or income) would alter the results. Pearson and Spearman correlations between percentage male-male households and all unmarried households vs percentage male-male households and all households were also calculated to evaluate whether there was significant difference in using all unmarried households or all households in our analysis.

Analysis was performed comparing census tracts with at least 1% male-male unmarried households with census tracts containing less than 1% male-male unmarried households. We repeated analyses using 5% and 10% cutoffs to assess whether the association held with increasing concentrations of male-male households. Specifically, we used cumulative binary variables, repeating analyses for neighborhoods with at least 1% (compared with those with <1%), at least 5% (compared with those with <5%), and at least 10% (compared with those with <10%) male-male unmarried households. To control for possible confounding by neighborhood socioeconomic characteristics, we also adjusted for median household income, percentage young female residents (defined as women aged 15-24 years), and percentage white, non-Hispanic residents at the census tract level. Census data were obtained from the 2016 American Community Survey (5-year estimates for 2012-2016), which is a smaller annual survey conducted by the US Census Bureau detailing population and housing information that helps local officials, community leaders, and businesses understand changes taking place in their communities.^12^

Finally, to account for the modifiable areal unit problem (ie, boundaries of census tracts, zip codes, school districts, and so on are arbitrary and do not define a person’s true neighborhood of exposure and influence),^13^ we calculated a 1-mile euclidean buffer around each tanning salon location and repeated the above analyses. Each census tract with at least 1 of these circular buffers was considered near a tanning salon location, with all others outside of tanning salon locations.

### Statistical Analysis

We first performed *t* tests investigating differences in mean percentage of male-male partnered households per census tract, with and without tanning salon locations within the census-tract boundary. We used binary logistic regression to determine the odds ratio of a census tract with male-male households above a prespecified percentage and containing at least 1 tanning salon location within all census tracts in our 10-city sample. We repeated *t* tests and binary logistic regression models with census tracts within and outside of 1 mile of a tanning salon location, and analyses were repeated after adjusting for various demographic characteristics of census tracts. In lieu of running 10 separate logistic models to obtain estimates within each city, we used a nonlinear mixed model with a random intercept grouped by city for the 10 cities to compare the neighborhood distribution of salons with the mean for the city in which it is situated rather than with the United States as a whole. All analyses were performed with SPSS version 25 (IBM Corp). Statistical significance was set at *P* < .05, and all tests were 2-tailed.

## Results

Across the 10 cities in this study, there were a total of 4091 census tracts. A total of 196 tanning salons were identified within 176 individual census tracts, with 0 to 3 tanning salons per census tract. There were 482 823 unmarried partnered households, of which 35 164 (7.3%) were male-male (median per census tract, 0; range, 0-392). The median (interquartile range) percentage of male-male partnered households per census tract was 0% (0%-10.6%). Overall, 1525 of 4091 census tracts (37.3%) had at least 1% male-male unmarried partnered households, 1373 (33.6%) had at least 5% male-male unmarried partnered households, and 931 (22.8%) had at least 10% male-male unmarried partnered households.

**Figure 1.**
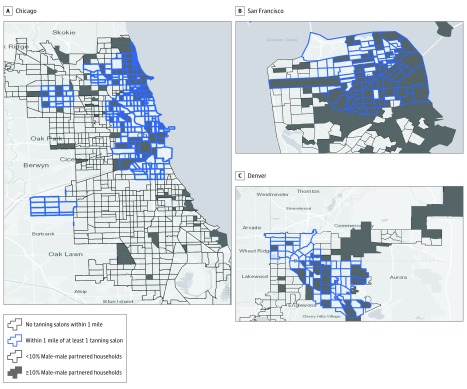
Association of Male-Male Partnered Households With Tanning Salon Locations in Chicago, Illinois, San Francisco, California, and Denver, Colorado A, Odds ratio of having a tanning salon within 1 mile of a census tract with more than 10% male-male partnership in Chicago was 3.77 (95% CI, 1.80-7.88). B, Odds ratio of having a tanning salon within 1 mile of a census tract with more than 10% male-male partnership in San Francisco was 1.88 (95% CI, 1.06-3.31). C, Odds ratio of having a tanning salon within 1 mile of a census tract with more than 10% male-male partnership in Denver was 2.20 (95% CI, 1.60-3.31).

**Figure 2.**
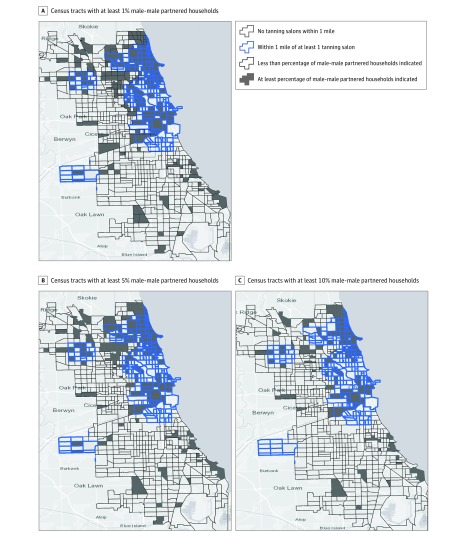
Percentage of Male-Male Partnered Households in Chicago, Illinois, and Census Tracts Within 1 Mile of a Tanning Salon A, Odds ratio of having a tanning salon within 1 mile of a census tract with more than 1% male-male partnership was 3.83 (95% CI, 2.50-5.76). B, Odds ratio of having a tanning salon within 1 mile of a census tract with more than 5% male-male partnership was 3.68 (95% CI, 2.56-5.27). C, Odds ratio of having a tanning salon within 1 mile of a census tract with more than 10% male-male partnership was 3.77 (95% CI, 1.80-7.88).

Results from logistic regression analyses showed that indoor tanning salons were more likely to be located in census tracts with higher proportions of male-male households. The odds of having an indoor tanning salon in a census tract with at least 10% male-male partnered households were significantly greater than in a census tract with less than 10% male-male partnered households (odds ratio [OR], 2.17; 95% CI, 1.59-2.97). This association was also statistically significant for Chicago, Denver, and San Francisco (Figure 1). The OR of a census tract with at least 5% male-male partnered households compared with a census tract with less than 5% male-male partnered households having an indoor tanning salon was 2.04 (95% CI, 1.51-2.77). At the 1% cutoff for male-male households, the OR was 2.36 (95% CI, 1.73-3.19). Figure 2 shows tanning salon locations and percentage of male-male partnered households based on these increasing thresholds in Chicago, which had the highest association between the 2 metrics.

The overall association of tanning salon location with male-male partnered households did not change substantially when adjusted for neighborhood socioeconomic status using median household income, percentage young women, and percentage white, non-Hispanic residents. Table 1 shows logistic regression data of the association of tanning salons with study variables, including SMM census tracts by percentage of male-male partnered households, adjusted for the 3 previously mentioned variables. For example, the odds of an indoor tanning salon being within 1 mile of a census tract with at least 10% male-male households, after adjusting for median household income, percentage young women, and percentage non-Hispanic white residents, remained 2-fold that of census tracts with less than 10% male-male households (OR, 2.00; 95% CI, 1.71-2.35).

**Table 1.** Correlates of Census Tracts With 1 or More Tanning Salons in the 10 US Cities With the Highest Lesbian, Gay, Bisexual, and Transgender Populations

Variable	β	*P* Value	Odds Ratio (95% CI)
Logistic model			
>1% Male-male households	0.62	<.001	1.85 (1.35-2.53)
>5% Male-male households	0.48	.002	1.61 (1.18-2.20)
>10% Male-male households	0.57	.001	1.77 (1.28-2.44)
Nonlinear mixed model			
>1% Male-male households	0.51	.002	1.67 (1.21-2.30)
>5% Male-male households	0.48	.02	1.46 (1.06-2.01)
>10% Male-male households	0.57	.004	1.63 (1.16-2.27)

The 1-mile buffer around each indoor tanning salon yielded 1364 (33.3%) census tracts located within 1 mile of a tanning salon location (compared with 176 census tracts with a tanning salon within their boundaries); all others (2727 census tracts) were not within 1 mile of an indoor tanning salon. Analyses were rerun using this buffer; the OR of a census tract with at least 10% male-male partnered households being within 1 mile of a tanning salon, compared with a census tract with less than 10% male-male partnered households, was 2.48 (95% CI, 2.14-2.88). The ORs of a census tract with at least 5% or 1% male-male partnered households being within 1 mile of a tanning salon compared with a census tract with less than 5% or 1% male-male partnered households were 2.58 (95% CI, 2.25-2.96) and 2.75 (95% CI, 2.40-3.14), respectively. Table 2 summarizes the analysis using this 1-mile buffer after adjusting for median household income, percentage young women, and percentage white non-Hispanic residents.

**Table 2.** Correlates of Census Tracts Within 1 Mile of 1 or More Tanning Salons in 10 US Cities With the Highest Lesbian, Gay, Bisexual, and Transgender Populations

Variable	β	*P* Value	Odds Ratio (95% CI)
Logistic model			
>1% Male-male households	0.78	<.001	2.18 (1.89-2.51)
>5% Male-male households	0.72	<.001	1.61 (1.18-2.20)
>10% Male-male households	0.70	<.001	2.00 (1.71-2.35)
Nonlinear mixed model			
>1% Male-male households	0.67	<.001	1.96 (1.69-2.27)
>5% Male-male households	0.62	<.001	1.86 (1.60-2.16)
>10% Male-male households	0.60	<.001	1.82 (1.54-2.15)

Results from a nonlinear mixed model used to compare the neighborhood distribution of salons with the intracity mean did not substantially differ from the logistic regression model (Table 1 and Table 2), with all 6 models still showing a statistically significant association between higher concentrations of male-male households within a census tract and the presence of a tanning salon. For example, the odds of an indoor tanning salon being within 1 mile of a census tract with at least 5% male-male partnered households was 1.86 (95% CI, 1.60-2.16) in the nonlinear mixed model, compared with 1.61 (95% CI, 1.18-2.20) in the logistic regression model.

A sensitivity analysis conducted by calculating the percentage of male-male partnered households as a fraction of the total number of households in each census tract (rather than as a fraction of the total number of unmarried partnered households) yielded 685 census tracts with more than 1% male-male households, 26 with more than 5% male-male households, and just 4 with more than 10% male-male households, of 4091 total census tracts. The Pearson 2-tailed correlation between percentage of male-male partnered households with unmarried households and percentage of male-male partnered households with total households was 0.79 (*P* < .001) (Spearman correlation, 0.984; *P* < .001). Within this sensitivity analysis, the odds of a census tract with at least 1% unmarried partnered male-male households having a tanning salon was 1.69 (95% CI, 1.20-2.39).

## Discussion

Our study shows that indoor tanning salons were more than twice as likely to be located in or near neighborhoods with a higher proportion of male-male partnered households. This association held even after adjusting for median household income, percentage young women, and percentage white non-Hispanic residents. Indoor tanning using UV radiation is a group 1 carcinogen, defined by the World Health Organization as being a substance carcinogenic to humans.^14^ Therefore, the study results have potentially important public health implications for SMM, given their higher risk of developing skin cancer.^2^ The presence of tanning salons close to communities at high risk for skin cancer raises questions about potential industry targeting of populations at higher risk of using indoor tanning.

The association of the presence of indoor tanning salons with neighborhoods with higher concentrations of gay men remained statistically significant when we performed sensitivity analyses varying our underlying analytic assumptions. For example, we expanded our sample to include census tracts within 1 mile of an indoor tanning salon, which may better approximate tanning salon location exposure, as it has been shown that boundaries of census tracts, zip codes, and school districts are arbitrary and do not define a person’s true neighborhood of exposure and influence.^12^ In addition, we calculated the concentration of male-male households in different ways and found that these measures were highly correlated and yielded similar conclusions. As described in the Methods section, to assess face validity of our measures, we confirmed that the neighborhoods with the highest concentrations of male-male households were in the Castro District of San Francisco and West Hollywood area of Los Angeles, well-known neighborhoods with high LGBT populations. This supports our method of using male-male unmarried partnered households as a proxy for areas with higher concentrations of gay men.

Increasing evidence suggests that certain industries may target the LGBT community, contributing to health disparities. For example, tobacco is a cancer risk factor that disproportionately affects LGBT people.^15^ Indeed, LGBT individuals are more than twice as likely to smoke tobacco compared with heterosexual adults, and research shows that the tobacco industry not only is aware of this disparity but may actively contribute to it by targeting LGBT communities through marketing campaigns, direct and indirect advertising, and community outreach.^16,17,18,19^ Alcohol companies, many either owned by or found to have direct alliances with tobacco firms, may also have targeted gay men as early as the 1950s.^20,21^

Similar to our findings regarding the geographic distribution of indoor tanning salons, prior research has shown a significantly higher tobacco retailer density in neighborhoods with higher concentrations of same-sex couples.^11^ This correlation is strongest in analyses of male-male couples, even after controlling for factors such as median household income and other area-level characteristics.^11^ A nationally representative survey showed that tobacco companies have targeted consumers online in new and social media, with LGBT individuals reporting significantly higher rates of past 30-day tobacco media exposure compared with non-LGBT individuals.^22^ Also, LGBT people more frequently reported exposure to, searching for, or sharing messages related to tobacco, tobacco couponing, e-cigarettes, and antitobacco campaigns on social media compared with non-LGBT people.^22^ The LGBT community is disproportionately targeted by advertising and is less likely to be included in tobacco control efforts.^23^

In qualitative focus groups with LGBT individuals, many viewed tobacco use positively, with targeting connoting community visibility, legitimacy, and economic viability. Participants did not view tobacco as a health issue specifically for LGBT individuals but also discredited the tobacco industry for lying. While most participants felt positive about inclusion in marketing, some differentiated this from targeting, which they saw as an unfortunate accompaniment to being included.^18^ In previous studies of focus groups with gay men, participants also expressed a strong dislike of the indoor tanning industry’s misinformation tactics, which is consistent with evidence from the literature about the tobacco industry and has been used by the antitobacco Truth campaign.^4,17,18^

Another study using geographic information systems found that a larger number of tanning facilities was associated with areas with greater percentages of young women.^8^ Additionally, observational studies have shown that indoor tanning facilities are prevalent on university campuses and in off-campus housing, consistent with data suggesting widespread use of indoor tanning among young adults in the United States.^24,25^ Furthermore, in some campuses, university-sponsored debit cards may be used to pay for tanning services, with 14.4% of colleges studied having such an agreement.^26^ These studies add to the evidence that indoor tanning may be more readily available to young adults at high risk and show that location and convenience may influence the prevalence of indoor tanning within groups at high risk of using these services.

### Limitations

This study has several limitations. First, there is no comprehensive list of tanning salon locations. We cross-referenced 2 databases to capture as many tanning salon locations as possible but could not include nonbusiness tanning beds that are not available on either of these platforms (eg, tanning beds in gyms, apartment complexes, college campuses, and hotels), which may skew our conclusions. Second, census data only provide information on same-sex partnered households, and data on individual sexual orientation were not available at the time of this study. Partnered SMM may exhibit different patterns in neighborhood selection and health behaviors, and thus, this research may not be generalizable to all SMM. Third, there is an 8-year gap between the latest available census data and the tanning salon data, which is long enough for some meaningful changes in neighborhood composition to have occurred—possibly leading to a slight underestimation of the calculated effects. Neighborhoods with increased male-male households between 2010 and 2018 may have attracted new tanning salons, while areas with declining male-male households may have lost salons. Additionally, spatial regression could not be run on the full model owing to the area between the 10 cities, and a spatial regression model was not powered enough to be run on each individual city. Specifically, we were not able to determine if the association between SMM and tanning salons varied across space within cities (eg, that the association was higher in neighborhood 1 and lower in neighborhood 2). We were only able to determine whether the association was significant across a contiguous city. We examined the 10 US cities with the largest concentration of LGBT people, and thus, this study may not be generalizable to other large LGBT populations. Finally, owing to the ecologic nature of this study, inferences about the behaviors of any individuals or populations cannot be drawn.

## Conclusions

Our study findings suggest that tanning salons were more likely to be located in or near neighborhoods with higher concentrations of male-male partnered households. This may contribute to the disproportionate use of indoor tanning by SMM and be a contributing factor to health disparities in skin cancer prevalence within this community.
